# Presentation and anatomical distribution of diverticular disease in four hospitals in Sudan

**DOI:** 10.11604/pamj.2020.36.64.22987

**Published:** 2020-06-03

**Authors:** Alsmwal Alnour Alnzaer, Ali Yasen Yasen Mohamedahmed, Yousif Abdalla Adam, Elmoiz Eltyiep, Suliman Hussen Suliman

**Affiliations:** 1 General Surgery, Sudan Medical Specialization Board, Khartoum, Sudan,; 2 Department of General Surgery, University of Khartoum, Khartoum, Sudan

**Keywords:** Diverticular disease, rectal bleeding, constipation

## Abstract

**Introduction:**

diverticular disease (DD) was thought to be more prevalent in the western countries, especially the white populations, but the recent increase in incidence among African and Asian population, was reported. Up to our knowledge, there is no previous study of DD in Sudan.

**Methods:**

this is a descriptive cross-sectional study conducted at the department of endoscopy in four Sudanese hospitals in the period from October 2017 to February 2019. We included all patients who underwent colonoscopy during the study period. The main objective is to study the presentation and the anatomical pattern of diverticular disease among the Sudanese population.

**Results:**

prevalence of DD in the included population was 7.5% (104/1393). The mean age was 66.4 ± 12.5 years with the percentage of males in our study is 77.1% and females were 22.9%. Presenting complains were: abdominal pain in all patients, constipation in 78.8% and rectal bleeding in 57.7%. Regarding anatomical distribution: 63.5% have left colonic DD, 19.2% in the right colon and 17.3% involving the entire colon. There was a significant correlation between the left side DD and following clinical presentations: mucus per-rectum (p = 0.015) and weight loss (p = 0.048). Other endoscopic findings of significance were internal pile in 21.2% and colo-rectal polyp in 15.4%.

**Conclusion:**

the prevalence of DD in the included population, is 7.5% which is consistent with recent literature from the Middle East, Africa and Asia but still less than the prevalence in the western countries and left side colon is predominantly affected.

## Introduction

Diverticular disease (DD) is a sac-like protrusion of mucosa through the muscular colonic wall. The protrusion occurs in weak areas of the bowel wall through which blood vessels (vasa recta) penetrate. Colonic diverticula are usually pseudo-diverticula, as they contain only mucosa and sub-mucosa covered by serosa [1-3]. The incidence of DD demonstrates a clear age dependency, although a slight increase in younger patients was observed during the last decade [4, 5]. Lack of dietary fibre, smoking, high body mass index (BMI), alcohol consumption and nonsteroidal anti-inflammatory drugs (NSAIDs) are firmly anchored to DD in the literature [6-10]. Moreover, the following rare syndromes demonstrate a strong predisposition for colonic diverticula formation; Marfan syndrome, Ehlers-Danlos syndrome, Williams-Beuren syndrome, Coffin-Lowry syndrome and polycystic kidney disease [5, 11, 12]. For many years it has been thought that DD exclusively affects westernized countries due to a lack of fibre in the diet, which leads to increase the pressure on the colonic wall [13]. However, recent data has revealed an increase in the prevalence of colonic diverticulosis throughout the world [14]. A low prevalence of diverticulosis was reported in patients who underwent colonoscopy in African countries; ranging from 2% to 13.5% [14-16]. However, a higher incidence was reported in Asian countries, which was up to 28.5% with predominantly on the right side [17, 18]. Much higher prevalence was reported in western countries (57.7% in the USA, 47% in the UK, 49% in Germany, 50% in Finland and 19.9% in Italy) and affecting the left colon in the vast majority of cases [19-23]. In light of this vast ethnicity variation and lack of reports from Sudan regarding pattern and anatomical distribution of DD, we conducted this study to evaluate the pattern of DD in the Sudanese population.

## Methods

This is a descriptive observational study that was conducted in the endoscopy department in four major Sudanese hospitals: Soba University Hospital, Ibn Sina Specialized Hospital, Omdurman Military Hospital and Alribat Teaching Hospital, in the period from February 2017 to February 2019. All the hospitals mentioned above have an endoscopy unit with consultant physicians and surgeons performing the endoscopy lists and most of the people who required colonoscopy in Khartoum State are referred to one of these hospitals. We include all patients who underwent colonoscopy irrespective if DD is suspected or not.

**Inclusion and exclusion criteria:** we include all patients attending the endoscopy units in the above-mentioned hospitals during the study period and had a colonoscopy to investigate gastrointestinal symptoms such as: changes in bowel habits, rectal bleeding, anaemia unexplained weight loss. Exclusion criteria were: patients with inadequate bowel preparations, an incomplete examination of the colon and patients who are unwilling to participate in the study. Patients that were confirmed to have DD on colonoscopy were studies to understand the possible risk factors and anatomical distribution. Rectal bleeding was considered major when it required admission to the hospital, such as association with systolic blood pressure less than 90, bleeding that required blood transfusion or persistence of bleeding for more than three days. Moderate rectal bleeding is three episodes or more of rectal bleeding (fresh or dark), or melaena in a haemodynamically stable patient. Two or fewer episodes of rectal bleeding without the previously mentioned criteria were considered as mild rectal bleeding. Data were analysed with statistical package for the social sciences (SPSS) version 23. Qualitative data were analysed using correlation test and simple linear regression and the P-value was considered significant if less than 0.05. Written informed consent obtained from each participant and ethical clearance was obtained from the ethical committee of Sudan Medical Specialization Board, as well as hospital administration approval.

## Results

**Figure 1:**
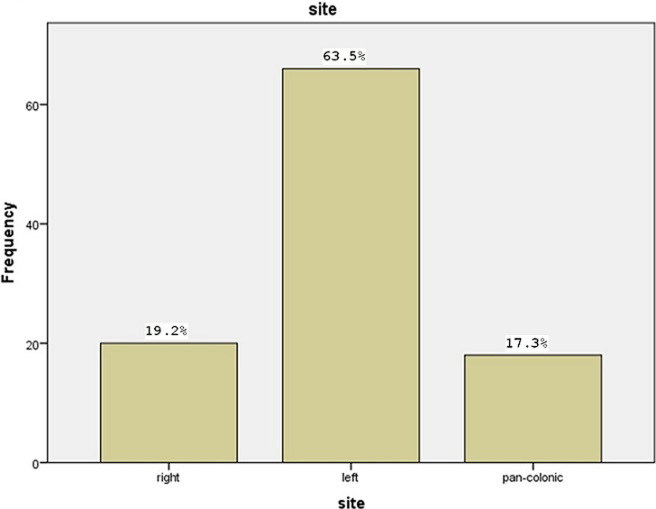
anatomical distribution of colonic diverticulosis

The total number of patients who underwent colonoscopy during the period of study were 1393 patients and 104 of them were confirmed to have DD with a prevalence of 7.5%. Furthermore, patients who confirmed to have DD were identified and studies for baseline characteristics, clinical presentation and risk factors. The mean age was 66.4±12.5 and 70.2% of patients were male. Baseline characteristics and risk factors for diverticulosis in the study population are shown in Table 1. Moreover, 61 out of all patients have medical comorbidities; hypertension was noted in 49.2%, diabetes was noted in 27.9% and 11.5% has ischaemic heart disease. Abdominal pain was the presenting symptom in all patients confirmed to have DD with left iliac fossa pain reported in 58.6%, right iliac fossa reported in 21.1% and generalized abdominal pain reported in 20.2%. In addition, rectal bleeding was the presenting symptom in 57.6% and the degree of bleeding were described as major in 9.6% of patients, moderate in 26% of patients and minor in 22.1% of patients. Table 2 shows clinical presenting symptoms. Regarding the anatomical distribution of colonic diverticulosis, more than half of the cases (63.5%) had left colonic diverticulae, 19.2% of patients have colonic diverticulae in the right colon and in 17.3% the disease involved the entire colon (Figure 1). Furthermore, the left side diverticulae showed a statistically significant higher risk for mucus per-rectum (P-value=0.015) and weight loss (P-value=0.048). However, there was no statistically significant risk between the site of diverticulae and the following outcomes: rectal bleeding (P-value=0.496), bowel habits (P-value=0.87), abdominal bloating (P-value=0.135), anaemic symptoms (P-value=0.519), anorexia (P-value=0.154) and constipation (P-value=0.207) (Table 3). Other endoscopic findings of significance were internal pile in 21.2% and colo-rectal polyp in 15.4%, other outcomes like colitis and masses occur less frequently (Table 4).

**Table 1:** shows demographic characteristics of included population and risk factors

Outcome		Number of patients (n=104)	Percentage
Age group	0-19	01	00.9%
	20-39	04	03.9%
	40-59	18	17.4%
	60-79	67	64.4%
	80-100	14	13.4%
Gender	Male	73	70.2%
	Female	31	29.8%
BMI	< 18.5	05	04.8%
	18.5-24.9	65	62.5%
	25-29.9	13	12.5%
	30-34.9	14	13.5%
	35-39.9	03	02.9%
	≥ 40	04	03.8%
Risk Factors	Smoking	55	52.9%
	NSAIDS	35	33.7%
	Low fibre diet		85.6%
	Age more 70 years	49	47.1%
	Medical comorbidities	61	58.6%
	Obesity ( BMI ≥30)	21	20.1%

**Table 2:** shows clinical presentation of included population

Presenting Symptom	Number of patients (N=104)	Percentage
Abdominal pain	104	100%
Rectal bleeding	60	57.7%
constipation	82	78.8
Anaemia	35	33.7%
change in bowel habit	35	33.7%
abdominal distension	53	51.0%
mucus per rectum	17	16.3%
Anorexia	10	09.6%
Weight loss	19	18.3%

**Table 3:** relationship between side of the diverticulosis and clinical presentation

Parameter		Left side colon (n=66)	Right side colon (n=20)	Pan-colonic (n=18)	P-value
Rectal bleeding	Yes	37	11	12	0.496
	No	29	9	6	
Mucous secretion	Yes	9	2	6	0.015
	No	57	18	12	
Weight loss	Yes	8	4	7	0.048
	No	58	16	11	

**Table 4:** other significant findings during the colonoscopy

Colonoscopy finding	Number of patients (n=104)	Percentage
Internal pile	22	21.2%
Colonic polyps	16	15.4%
Colitis	04	03.8%
Colo-rectal mass	04	03.8%
Rectal ulcer	02	01.0%
Angiodysplasia	01	01.0%
Hirschsprung disease	01	01.0%
Stricture	01	01.0%
Redundant sigmoid colon	01	01.0%
No other finding	52	50.0%

## Discussion

Diverticular disease (DD) was thought to be more prevalent in the western world especially the white populations, but the recent increase in incidence among the immigrants from Africa who reside in the west for more than ten years may indicate that the westernization of the lifestyle could be the cause for this change in DD epidemiology [1, 23, 24]. In Africa, the Middle East and Asia the prevalence is less [14-18, 25-27]. In this study the prevalence of DD was 7.5% (104/1393) among all patients who underwent a colonoscopy to investigate gastro-intestinal symptoms which is comparable to a prevalence of 7.4% that reported previously in Saudi Arabia [27]. However, a higher prevalence of 13.5% and 12.1% were reported in studies from South Africa and North Korea, respectively [26, 28]. The prevalence in western countries is much higher 47% in the UK and up to 49% in Germany [21, 22]. The mean age was 67 ± 12.7 in this study which is similar to the reports from Africa and USA [16, 19, 27] but far different from Asian reports from Saudi Arabia with a mean age of 60.82 ± 0.833 and North Korea with a mean age of 50.9 ± 12.3 [27, 28]. The youngest patient in our study was 17 years of age. The youngest patient with colonic diverticulosis was described by Ignacio RC Jr who reported a 9 years old patient with William syndrome presented with rectal bleeding secondary to sigmoid diverticulitis [29].

The percentage of males in our study is 77.1% and females were 22.9% which is consistent with previous studies from Africa, the USA and Europe [16, 19, 27]. Risk factors for colonic diverticulosis include: low fibre diet, increased age, smoking, obesity, lack of exercise and certain medications (NSAIDs, anticoagulants and corticosteroid) [1, 15, 19, 25, 27, 28]. Sixty one patients were found to have one or more medical comorbidity in this study. Hypertension was found to be associated with increased risk of bleeding from DD, which is predominantly due to vascular endothelial injury and atheroma formation that leads to arteriosclerosis and increased pressure within exposed blood vessels which elevates the risk for bleeding [27]. A fifth of the included population (20.1%) showed a degree of obesity. However, there was no significant relation between obesity and rectal bleeding in this study (P-value=0.09). Several reports have shown that obesity increased the risk of DD complications, including rectal bleeding [30, 31]. A study from Sweden reported that obese patients with diverticular disease have a higher risk of hospitalization [32]. This may be due to the suggested link between obesity and inflammation along with intestinal flora differences in obese population [33]. Patients with DD can present with non-specific abdominal complaints, e.g. left-sided lower abdominal pain.

Patients with DD do not usually manifest signs of inflammation, such as pyrexia or neutrophilia, which could indicate diverticulitis. Pain is generally exacerbated by eating and diminished with defecation or flatus, suggesting colonic wall tension due to raised intraluminal pressure. Complicated DD presents according to the nature of the complication, which is predominantly abscess formation, perforation, Fistulation, bleeding, stricture or obstruction. Abscess formation is the most common presentation of acute complicated diverticulitis which occurs in approximately 15% of patients [1, 2]. A significant localized abscess may progress into a free perforation in 1-2% of patients with DD, which is associated with high morbidity and mortality [1, 2]. All patients in this study complained of abdominal pain at some point of the disease course. While the risk of bleeding from colonic diverticulosis is known to be around 15%; in this study, more than half the patients have rectal bleeding and 33.7% of patients were confirmed to have symptomatic anaemia. The mechanism of DD bleeding is thought to be due to rupture of the vasa recta at the neck of the diverticulum [34]. However, diverticular bleeding stops spontaneously in 90% of patients, there is a high risk for recurrence most commonly in patients with bilateral diverticulosis, on NSAIDs or anticoagulants [35-37].

In this study, we found that there is no statistically significant association between the degree of bleeding and smoking (p=0.526), NSAIDs (p=0.132) or different medical comorbidities (p=0.248). There is a clear overlap of symptoms between DD and different colonic pathologies, e.g. inflammatory bowel disease (IBS) and malignant tumours, the reason why a colonoscopy is becoming the gold standard investigation in patients with lower gastro-intestinal symptoms [1, 2, 23]. The anatomical site of the diverticula in this study was left site predominance with a percentage of 61.4% and this is consistent with studies from Europe and the USA [38]. However, reports from the Middle East and Africa revealed that the right side involved more frequently [1, 23, 27, 28, 38-41]. Association between colonic adenoma and DD was proposed by several authors as both shared common epidemiological trends and risk factors such as increased age and low fibre diet [42, 43]. An increased risk of 1.7-fold and 2.3-fold for colonic polyps in patients with DD were revealed among Japanese and African-American population respectively [44, 45]. In this study, 15.4% of patients with the diverticular disease were found to have colonic polyps.

## Conclusion

The prevalence of DD was 7.5% in patients who underwent a colonoscopy to investigate lower gastro-intestinal symptoms in this study, which is consistent with recent literature from the Middle East, Africa and Asia but still less than the prevalence in the western countries. The most common presentation is abdominal pain and rectal bleeding. The diverticular disease in this study is mainly left-sided which is not similar to reports from other countries in Asia and Africa where right side diverticular disease predominate. More studies are needed to investigate the outcome of treatment in the Sudanese population.

### What is known about this topic

- No published research about presentation and anatomical distribution of DD in Sudan;
- Prevalence of DD is rising in Africa.

### What this study adds

- The prevalence of the diverticular disease in the included population is 7.5%;
- Most common presentation in the study population is abdominal pain and rectal bleeding;
- The diverticular disease in the included population is mainly left-sided.
